# Recurrent infective endocarditis due to probable biofilm formation on cardiac stimulator probe

**DOI:** 10.1186/1471-2334-13-S1-P50

**Published:** 2013-12-16

**Authors:** Alina Cristina Neguț, Anca Streinu-Cercel, Maria Magdalena Moțoi, Luminița Bradu, Ioana Berciu, Oana Streinu-Cercel, Adrian Streinu-Cercel

**Affiliations:** 1 Carol Davila University of Medicine and Pharmacy, Bucharest, Romania; 2 National Institute for Infectious Diseases “Prof. Dr. Matei Balş”, Bucharest, Romania

## Background

The global increase in antibiotic resistance and the high number of implant-associated infections have rendered antibiotics ineffective in certain cases. So it’s time for a new therapy. Bacteriophages are obligatory intracellular parasites of bacterial cells and bacteria can be infected by bacteriophages. They have specificity of infection, due to the presence of specific receptors on the bacterium surface.

## Case report

We report the case of a 72 year-old male patient with recurrent infectious endocarditis with *Staphylococcus* spp on an endocavitary stimulation probe and grade III atrioventricular block. The patient had a dual chamber endocavitary cardiac VDD stimulator. The first probe was inserted through the left supraclavicular region. Because of a skin infection in this region, the probe was changed to the right supraclavicular region. During extraction, a fragment of the first probe remained inclavated in the right atrium.

In February 2012 the patient had the first episode of fever and chills and he received treatment with vancomycin for 5 weeks with good results. After 8 days, fever and chills reappeared. The patient was admitted to our hospital where he started treatment with linezolid and bacteriophages. After 1 month the same symptoms reappeared, and the patient received the same treatment. He remained afebrile for 2.5 months. The patient had positive hemocultures with different species of *Staphylococcus* during these episodes. The following episodes were treated with tigecycline and ceftaroline, but relapse occurred usually after 14-20 days of stopping antibiotic therapy.

Because of the frequent relapses, surgical removal of the probe remained the only viable option. The patient was admitted in a cardiovascular clinic, and under antibiotic protection they tried to remove the probe. Because of extensive fibrosis around the probe, extraction could not be performed, and during surgery they noticed a large, friable vegetation, with high risk of rupture. Because of severe cardiac failure the patient didn’t survive.

## Conclusion

The recurrent infection was probably due to biofilm formation on the cardiac probe and infection could not be medically eradicated, despite the use of antibiotics active on biofilm. The time span between relapses was longer when the patient received combined therapy, with phages and antibiotics. In such difficult to treat cases, it is important to set feasible objectives; maintaining quality of life and lengthening the interval between relapses can be, in the absence of a surgical cure, the best available option.

